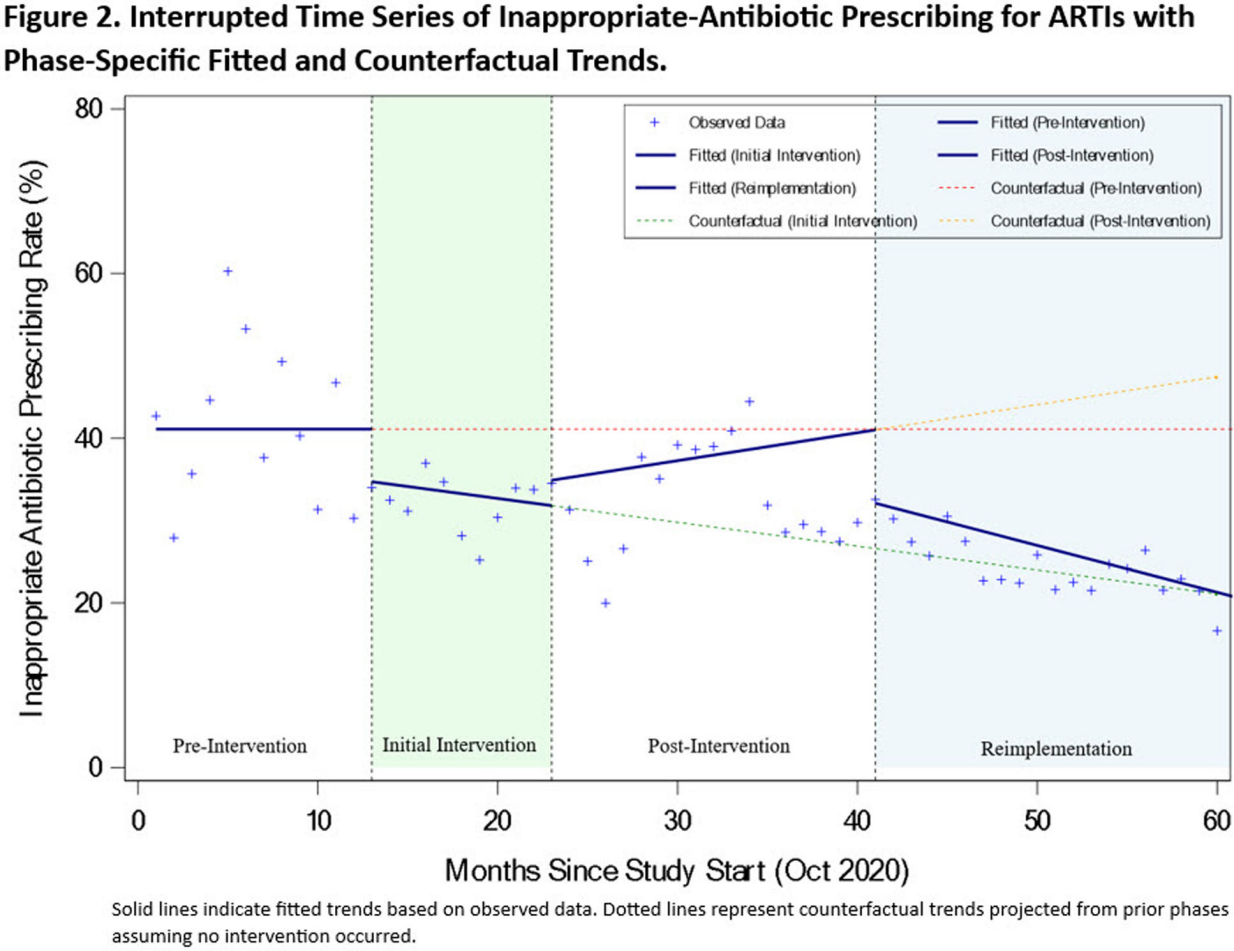# 81 Economic Burden of Unnecessary Outpatient Antibiotic Prescribing in Tennessee, 2022

**DOI:** 10.1017/ash.2026.10508

**Published:** 2026-06-23

**Authors:** Theresa Ubano Perez, Leslie Ann Loza-Zamora, Russel Grant, Massoud Dezfuli

**Affiliations:** 1 Eisenhower Health; 2 Eisenhower Healthcare Center

## Abstract

**Background:** Sustaining reductions in inappropriate antibiotic prescribing for acute respiratory tract infections (ARTIs) remains challenging, with improvements often diminishing after stewardship interventions end. In a large, rural, non-profit, healthcare system with three high-volume urgent care centers (UCCs), an initial 11-month behavioral intervention reduced inappropriate prescribing for ARTIs; however, rates increased after intervention cessation. We evaluated whether reimplementing the same intervention could sustain reductions in inappropriate antibiotic prescribing in urgent care settings. **Methods:** We conducted a quasi-experimental interrupted time series (ITS) analysis using monthly data from October 2020 through October 2025. The reimplementation of monthly individualized feedback and quarterly blinded peer comparison emails began in February 2024. Prescribing trends were analyzed across four phases: Pre-Intervention (October 2020 - September 2021), Initial Intervention (October 2021 - August 2022), Post-Intervention (September 2022 - January 2024), and Reimplementation (February 2024 - October 2025). Mean prescribing rates were compared using descriptive statistics and analysis of variance (ANOVA). A Seasonal Autoregressive Integrated Moving Average (SARIMA) model accounted for autocorrelation and seasonality. Analyses were performed using SAS 9.4 (SAS Institute Inc., Cary, NC, USA). **Results:** Among 52,842 ARTI visits, 15,180 antibiotics were prescribed during the study period. Mean inappropriate antibiotic prescribing decreased from 41.7% (SD 9.79%) Pre-Intervention to 32.3% (SD 3.36%) during Initial Intervention, followed by an increase to 32.6% (SD 6.58%) Post-Intervention, and subsequent decline to 24.4% (SD 3.79%) during Reimplementation (Figure 1). ANOVA revealed significant differences across phases (F(3,57)P P PPPPP **Conclusion:** Reimplementation of a behavioral stewardship intervention was associated with a larger and sustained, though non-significant, reduction in inappropriate antibiotic prescribing for ARTIs. These findings suggest that ongoing or repeated stewardship efforts may be necessary to maintain prescribing improvements in outpatient urgent care settings.

**Figure:**
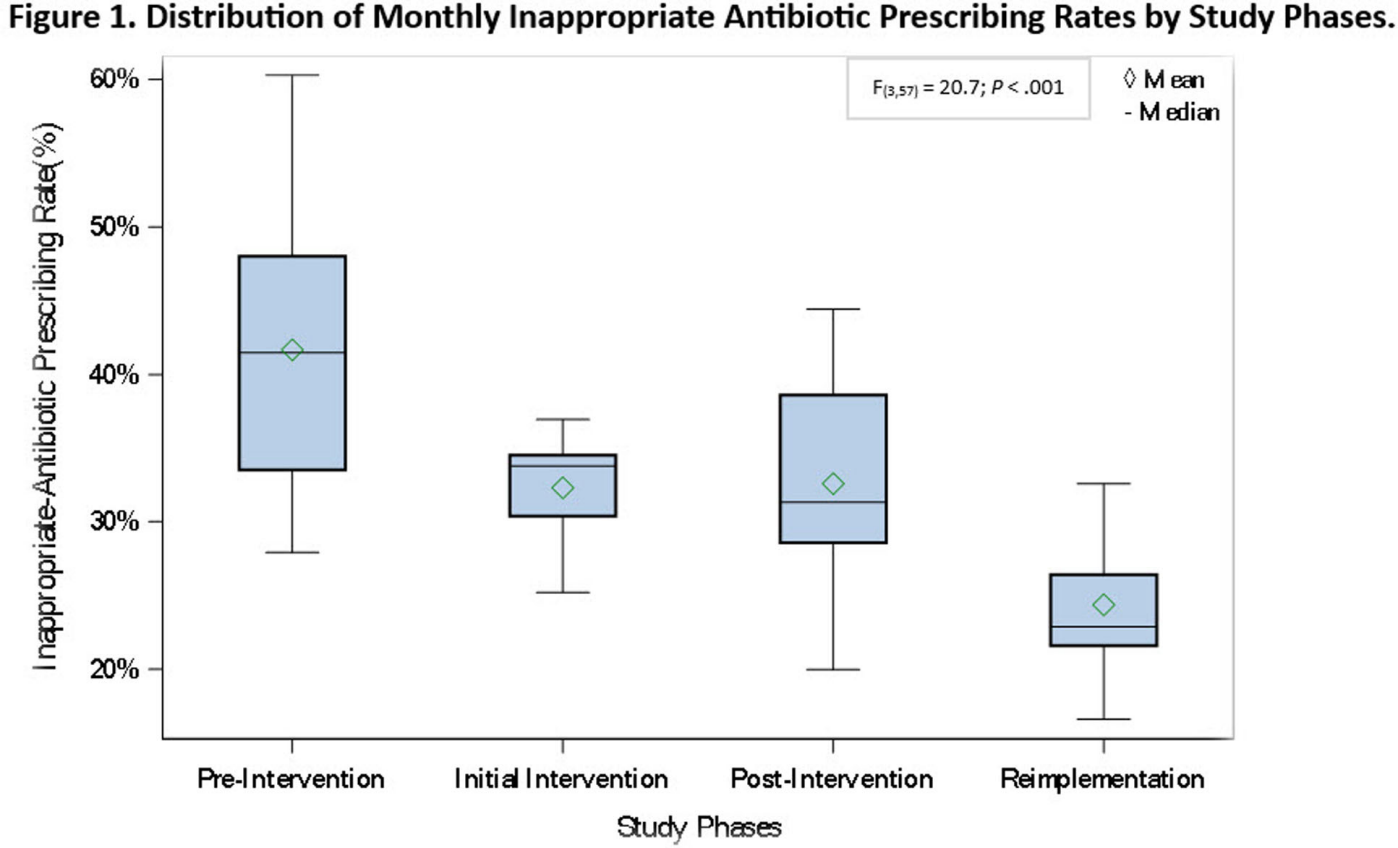

**Figure:**